# Advancements in molecular imaging for the diagnosis and treatment of pancreatic ductal adenocarcinoma

**DOI:** 10.1039/d4na01080a

**Published:** 2025-04-22

**Authors:** Xun Hu, Zihua Wang, Yuting Zhu, Zhangfu Li, Hao Yan, Xinming Zhao, Qian Wang

**Affiliations:** a Department of Diagnostic Imaging, National Cancer Center/Cancer Hospital, Chinese Academy of Medical Sciences and Peking Union Medical College Beijing 100021 China wangqian@cicams.ac.cn xinmingzh2017@yeah.net; b School of Basic Medical Sciences, Fujian Medical University Fuzhou 350122 Fujian Province China; c Department of Oral and Maxillofacial Surgery, Peking University Shenzhen Hospital Shenzhen Guangdong 518036 China; d Tsinghua Shenzhen International Graduate School/Tsinghua University Shenzhen 518055 China hyan23@sz.tsinghua.edu.cn

## Abstract

Pancreatic ductal adenocarcinoma (PDAC) is a highly malignant tumor characterized by poor overall patient survival and prognosis, largely due to challenges in early diagnosis, limited surgical options, and a high propensity for therapy resistance. The integration of various imaging modalities through molecular imaging techniques, particularly multimodal molecular imaging, offers the potential to provide more precise and comprehensive information about the lesion. With advances in nanomedicine, new imaging and drug delivery approaches that allow the development of multifunctional theranostic agents offer opportunities for improving pancreatic cancer treatment using precision oncology. Herein, we review the diagnostic and therapeutic applications of molecular imaging for PDAC and discuss the adoption of multimodal imaging approaches that combine the strengths of different imaging techniques to enhance diagnostic accuracy and therapeutic efficacy. We emphasize the significant role of nanomedicine technology in advancing multimodal molecular imaging and theranostics, and their potential impact on PDAC management. This comprehensive review aims to serve as a valuable reference for researchers and clinicians, offering insights into the current state of molecular imaging in PDAC and outlining future directions for improving early diagnosis, combination therapies, and prognostic evaluations.

## Abstract 2

Application of molecular imaging techniques for diagnosis and treatment of PDAC.

## Graphical abstract

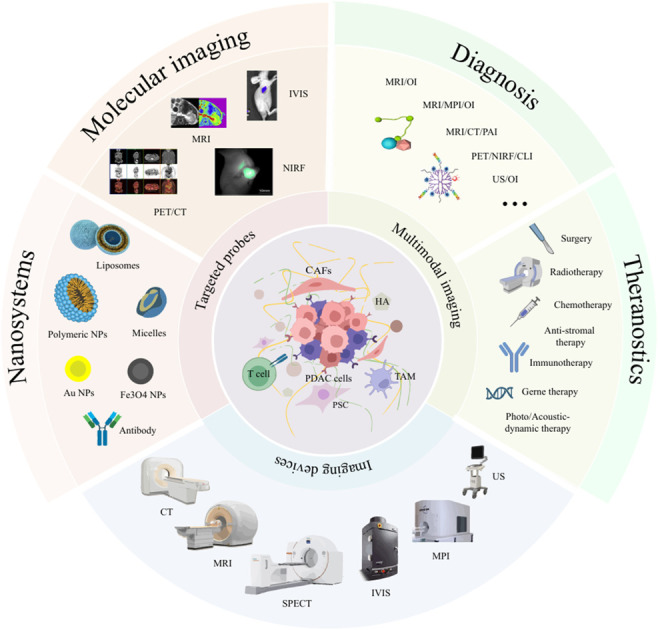

## 1. Introduction

Pancreatic ductal adenocarcinoma (PDAC) is one of the diseases with the poorest prognoses, which causes nearly 5% of global cancer deaths and has an overall 5-year survival rate of less than 5%.^1^ Conventional serological and imaging approaches are not always effective in diagnosing the disease in its early stages due to its insidious characteristics, and the lack of specific symptoms and effective biomarkers in the early stage. Over 85% of patients with PDAC are at a distant metastasis or locally advanced stage at the time of diagnosis, missing the chance for surgery.^2^ Chemotherapy is the first-line treatment option for inoperable patients recommended by pancreatic cancer treatment guidelines, with the FOLFIRINOX multiplex regimen and the doublet regimen of gemcitabine(GEM) and albumin-bound paclitaxel as the primordial choice.^3^ Additionally, the survival prognosis of individuals with pancreatic cancer has not been dramatically and considerably improved by particular antibodies, Immune Checkpoint Therapy (ICT), or Adoptive Cell Transfer Therapy (ACT). The overall survival rate of patients with PDAC increased from just 2% in 2012 to 11% in 2022, despite pharmaceuticals licensed by the U.S. Food and Drug Administration (FDA).^4^ The diagnosis and treatment of pancreatic cancer remain mired in the embarrassing “difficult to diagnose early, few interventions, resistant to drugs, and poor prognosis” state. Accurate treatment response assessment and early diagnosis are critical for prompt clinical decision-making.

Clinical imaging of PDAC relies on standardized procedures. Diagnostic efficacy depends on the doctor's expertise, instrument availability and clinical symptoms. Computed tomography (CT), magnetic resonance imaging (MRI), positron emission tomography (PET), single photon emission computed tomography (SPECT), ultrasound (US) and rndoscopic retrograde cholangiopancreatography (ERCP) are clinical diagnostic imaging modalities that are frequently employed for pancreatic tumors. Every imaging modality has specific benefits and drawbacks (Table 1). For example, MRI can capture physiological and anatomical details without ionizing radiation, but it also has long imaging times, contraindications, and nephrotoxicity.^5,6^ Similarly, PET/SPECT can image biochemical processes but has low resolution and carries a radiation exposure risk. Although existing imaging techniques can provide morphological information about pancreatic tissue, unimodal imaging has low sensitivity and specificity for the diagnosis of pancreatic cancer. There are numerous obstacles still facing early clinical monitoring of PDAC. Pancreatic cancer is not sensitive to conventional chemoradiotherapy. Therefore, it is urgent to find early diagnostic means and effective treatment for pancreatic cancer.

**Table 1:** Clinical selected modalities in PDAC and relative advantages and disadvantages

Modality	Advantage	Disadvantage	Uses in PDAC
US	①Non-radiation and non-invasive	①Low resolution	Screening
	②Flexible and low cost	②Dependent on operator skills	
	③Multi-view real-time imaging	③Influenced by gases and patients	
	④Guided intervention		
CT	①High spatial resolution	①Qualitative skills	Pancreas imaging preferred
	②Low radiation	②Tissue non-specific	
	③Enhanced scanning		
MRI	①Non-radiation and non-invasive	①High cost and time	Complementary imaging for CT
	②Excellent soft tissue imaging	②Artifact interference	
	③Multi-parameter, multi-view imaging	③Many contraindications	
	④Visualisation of the pancreas and bile ducts	④Nephrotoxicity	
PET/SPECT	①Early monitoring of efficacy	①Radioactive radiation	Excellent imaging of lymph nodes and distant metastatic lesions
	②Providing metabolic information	②Low resolution	
		③High cost	
EUS	Identifies thw tumour stage	①Invasive	Acquisition of tissue specimens
		②Dependent on operator skills	
ERCP	①Effective in those with obstructions or abnormal changes in the lower biliary tract and pancreatic ducts	Unable to display tumours directly	Patients with inoperable obstructive jaundice
	②Acquisition of tissue specimens		

Traditional structural imaging methods are evolving into functional and molecular imaging as medical imaging technology develops. These approaches can reveal to us the biological characteristics of tumors and cell changes that occur during treatment. Molecular imaging, which emerged at the end of the 20th century, is a multidisciplinary technique that has proliferated because of the development of animal models of human diseases and ongoing advancements in imaging equipment technology. It can non-invasively monitor biochemical processes and target localization that are not detectable by anatomical imaging methods,^7^ increasing the sensitivity and accuracy of tumorigenesis detection and providing the opportunity for early tumor identification. Molecular imaging methods can be separated into two main groups:, imaging hardware with excellent spatial and temporal resolution and highly selective and sensitive contrast agents like certain targeted probes.^8,9^ Nanoparticles are widely used in tumor diagnosis and treatment because of their unique physical and chemical properties such as the size effect, good biocompatibility and easy surface modification. The characteristic coupling of a variety of specific contrast agents, therapeutic drugs, targeted molecules, *etc.* with nanoparticles can not only prolong the half-life of contrast agents, improve the single imaging mode, and improve biocompatibility, but also reduce the toxic side effects of therapeutic drugs, and realize the integration of diagnosis and treatment of pancreatic cancer. This article concentrates on novel developments and clinical applications of specific probes, particularly nanoparticle probes, in the early diagnosis and combination treatment of PDAC.

## 2. Classical molecular imaging probes

### 2.1 Magnetic resonance imaging

Because of its superior soft tissue contrast and radiation-free characteristics, MRI has emerged as a crucial tool for the early detection and treatment monitoring of PDAC. The principle is to create a specific magnetic field around the human body, and then detect the transverse and longitudinal relaxation signals produced by the hydrogen nuclei in the body when electric radiofrequency pulses activate them. The process by which the transverse magnetization gradually decreases is called transverse relaxation, or T_2_ relaxation. The process by which the longitudinal magnetization is gradually restored is called longitudinal relaxation or T_1_ relaxation.^10^ While nanoscale superparamagnetic iron oxide nanoparticles (SPIO) can be employed to shorten T_2_ relaxation time (negative contrast agents), gadolinium-chelated contrast agents, such as Gd-DTPA, are frequently used in medical imaging, primarily to enhance T_1_ signal intensity. Advances in molecular imaging techniques drive MRI to enable imaging of the tumor microstructure and biomarkers. Significant advances have been made in MRI for the targeted imaging of PDAC, depending on the identification of specific biomarkers (Table 2). Liu *et al.*^11^ designed an anti-mesothelin antibody-modified nanoprobe (Fe_3_O_4_@SiO_2_) that enables specific targeting of pancreatic cancer cells. This nanoprobe showed a high degree of stability and biocompatibility in *in vitro* experiments. Utilizing Siemens 3.0 T MRI equipment, after injection of the probe, the tumor signal was reduced in the experimental group, while that in the control group remained almost unchanged. The mucin (MUC) family, a group of highly glycosylated macromolecules, are considered promising therapeutic targets. Zou *et al.*^12^ developed MUC1-SPIONs that specifically target mucin 1. MUC1-SPIONs also demonstrated the capacity to target pancreatic cancer cells in MRI imaging specifically. Wang *et al.*^13^ synthesized enolase 1-targeted SPIO(ENO-1) nanoparticles and confirmed their efficacy in the specific detection of PDAC. These results suggest that specific targeting probes can enhance the sensitivity of PDAC detection.

**Table 2:** Molecular probe targets for PDAC imaging diagnostics

Target	Imaging modalities	Type (Family)	Probe	Reference
MSLN	MRI	Membrane-anchored forms (mesothelin)	Fe_3_O_4_@SiO_2_	11
MUC1	MRI	Glycoprotein (epithelial barrier and cell signaling protein)	MUC1-SPIONs	12, 50 and 54
	MRI/OI		MN-EPPT	
	MRI/OI/MPS/PAI		RA-96 liposomes	
MUC4	MRI/OI	Glycoprotein (epithelial barrier and cell signaling protein)	MnMEIO-silane-NH2-(MUC4)-mPEG	49 and 60
ENO-1	MRI	Glycolytic enzyme (enolase)	ENO1-Dex-g-PCL/SPIO	13
Type I collagen	MRI	Group I collagen (collagen superfamily)	CM-101	20
EDB-FN	MRI	Fibronectins (extracellular matrix regulatory proteins)	MT218	21–23 and 51
	MRI/OI		ZD2-Gd-DOTA-Cy7	
IGF-1R	SPECT/CT	Receptor tyrosine kinases (insulin like growth factor)	^89^Zr-Df-1A2G11	27
Integrin αvβ6	PAI/OI	Transmembrane receptor (integrin family of cell adhesion receptors)	A740-R01	39
	SPECT/CT		^99^mTc-isoDGR	30
TROP2	SPECT/CT	Glycoprotein (TACSTD family)	^68^Ga-NOTA-RTD01	31
VEGF	MRI	Tyrosine kinase receptor (VEGFR family)	Bi50	61
EGFR	MRI	Tyrosine kinase receptor (ErbB family)		
	SPECT/CT		89Zr-MEHD7945A	28
CEA	MRI	Glycoprotein (immunoglobulin superfamily)	IONPs-PEG-MCC triple scAbs	38 and 60
			NbCEA5-ZW800-1	
CD44v6	MRI	Cell-surface receptor (CD44 antigen)	IONPs-PEG-MCC triple scAbs	60
GPC-1	MRI/OI	Proteoglycan (Glypican)	Gd-Au-NC-GPC-1	42 and 62
	NIRF/MRI		GPC1-GEM-NPs	
uPAR	MRI/OI	GPI-anchored receptor (plasminogen activation system)	ATF-IO	43 and 44
			DGL-U11	
Survivin gene	MRI/OI	Component of a chromosome passage protein complex (Baculoviral IAP repeat-containing)	Sur-MNPs	47
Plectin-1	MRI/OI	Structural component of muscle (Plakin)	Plectin-SPION-Cy7	45, 46 and 53
			Gd-Cy7-PTP/RGD	
	MRI/MPI/OI		PTP-Fe_3_O_4_-IRDye800CW	
CD326	MRI/OI	Glycoprotein (transmembrane glycoprotein EPCAM/Trop-2)	UCNP@Gd@PEG-CD326	48
LDLR	PAI/OI	Receptor (low-density lipoprotein receptor)	Peptide-22-Cy7	56
ICAM-1	PET/OI/CLI	Ligands for the leukocyte adhesion protein LFA-1 (integrin alpha-L/beta-2) (Intercellular adhesion molecule/vascular cell adhesion molecule)	[^89^Zr] Zr-DFO-ICAM-1-IR800	57
CA19.9	PET/OI	Carbohydrate antigen	^ss^dual-5B1	58
Galectin-1	MRI/SPECT–CT/Handheld gamma camera	Lectin (Galectin-like)	^67^Ga-DOTA-*t*-PApep.1_LAC_-2000-MNP	59
HER2	MRI/OI	Tyrosine kinase receptor (ErbB family)	HER-PGFIO	63

The effectiveness and prognosis of cancer treatment can be predicted by tracking the urokinase-type fibrinogen activator system (uPA) activity. GR-4Am-SA,^14^ a novel non-metallic chemical exchange saturation transfer (CEST) MRI contrast agent, can potentially measure uPA activity *in vivo*. With a reduced CEST signal at 5.0 ppm and a salicylic acid moiety that generates a CEST signal at 9.5 ppm, the contrast agent contains peptides that can be cleaved by uPA. The two CEST signals can be used to characterize the amount of cleavage catalyzed by the enzyme in a single reaction coordinate.

PDAC has evolved into a unique tumor microenvironment (TME) that includes cancer-associated fibroblasts (CAFs), pancreatic stellate cells (PSCs), regulatory T-cells, tumor-associated macrophages (TAMs), and a large amount of extracellular matrix (ECM) rich in hyaluronic acid, fibronectin, chemokines, cytokines, and extracellular proteases.^15^ On the one hand, PDAC is genetically diverse, with a low mutational load and few neoantigens, resulting in low PDAC antigenicity. On the other hand, extracellular matrix aggregation, such as hyaluronic acid and collagen, increases solid stress and tissue interstitial hydraulics and compresses the tumor vascular system, leading to a low perfusion posture. Meanwhile, CAFs in PDAC's mesenchymal component modify the extracellular matrix and immunological microenvironment by secreting stroma and cytokines that promote tumor growth, invasion, and metastasis.^16^ Based on this, some studies^17–19^ have focused on targeting the pancreatic tumor stroma to disturb its dense structure or on modifying the immune microenvironment to improve treatment effects. An MRI probe (CM-101),^20^ which targets collagen-rich collagen in pancreatic cancer stroma, was able to selectively bind collagen and monitor fibrotic changes following treatment. Compared to the usual contrast agent Gd-DOTA, CM-101 showed a significantly improved signal in fibrotic tumor regions and an increased signal in tumors following chemotherapy. Extradomain-B fibronectin (EDB-FN), another tumor-associated protein in the stroma, predicts pancreatic cancer metastasis. MRI imaging with an EDB-FN-specific gadolinium-based contrast agent (MT218)^21,22^ successfully detected metastases and their surrounding tissue. There is also a dextran–peptide conjugate^23^ targeting EDB-FN, consisting of an antimagnetic and biocompatible dextran and a targeting peptide, and the dextran can be directly detected by CEST.CM-101 and MT218 are contrast compounds that target specific tumor stromal components and can provide comprehensive imaging of tumor tissues and metastases as well as monitor biological changes following treatment. The stability and specificity of these molecular markers provides fresh prospects and methodologies for molecular imaging of PDAC.

Molecular targeted probes, in conjunction with MRI, present a promising method for PDAC detection. Compared to conventional imaging techniques, this technology can yield more precise information, which is essential for early pancreatic cancer detection, evaluating the effectiveness of treatment, and tracking the development of the disease. MRI will be more beneficial for targeted imaging of PDAC if more specific biomarkers are found and new probes are consistently developed. Future research is required to confirm the safety and effectiveness of these probes in clinical settings and to determine whether or not they could be helpful in treating PDAC.

### 2.2 Nuclear medicine imaging

**Fig. 1:**
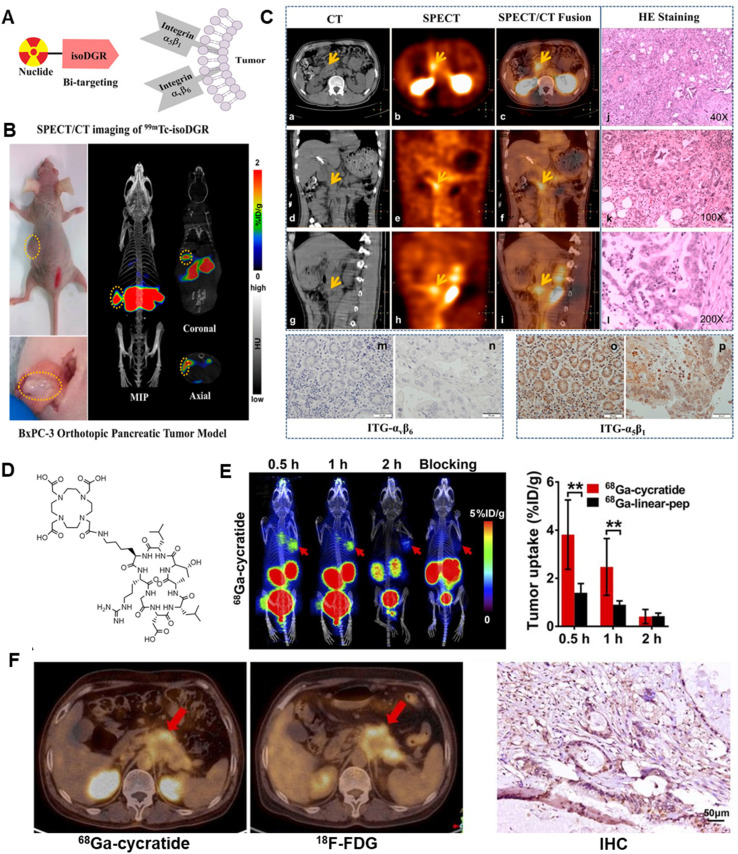
Nuclear medicine imaging based on diagnosis of PDAC. (A) Schematic structure of ^99^mTc-isoDGR; (B) representative small-animal nanoScan SPECT/CT images of BxPC-3 pancreatic cancer tumor-bearing mice; (C) SPECT/CT imaging of ^99^mTc-3PisoDGR2 in a 50-year-old man with moderately to poorly differentiated pancreatic adenocarcinoma; (D) chemical structure of DOTA-cycratide; (E) representative small-animal SPECT/CT images in BxPC-3 pancreatic cancer tumor-bearing mice; (F) PET/CT images of a female patient with suspected pancreatic cancer and her immunohistochemical (IHC) staining for integrin αvβ6.

Nuclear medicine imaging techniques, such as SPECT and PET, have shown significant potential in molecular imaging research and clinical applications for pancreatic cancer^24^ (Fig. 1). Common SPECT probes include labeled antibodies, peptides, and small molecules. For example, ^99m^Tc-labeled antibodies and peptide probes have been used in imaging studies of pancreatic cancer.^25^ SPECT probes are relatively stable, and the imaging equipment is more widespread and cost-effective. However, the drawbacks are lower spatial resolution and sensitivity compared to PET. PET offers high sensitivity and high spatial resolution, enabling early detection of small lesions. ^18^F-FDG is the most commonly used PET probe, but it also accumulates in inflammation and other benign conditions, leading to lower specificity in pancreatic cancer.^26^ The insulin-like growth factor-1 receptor (IGF-1R) is overexpressed in pancreatic cancer, and ^89^Zr -labeled IGF-1R probes have shown potential for imaging pancreatic cancer.^27^ The epidermal growth factor receptor(EGFR) is highly expressed in pancreatic cancer cells, and 89Zr-labeled anti-EGFR monoclonal antibodies have been used in PET imaging studies.^28^ Currently, various novel nuclear medicine probes are undergoing clinical trials to evaluate their effectiveness and safety in diagnosing pancreatic cancer. For example, ^68^Ga-RGD is currently under clinical investigation.^29,30^ Despite some challenges, with the development of new probes and the application of multimodal imaging technologies, nuclear medicine imaging will play an increasingly important role in the early diagnosis, treatment monitoring, and prognosis evaluation of pancreatic cancer. With PET/MRI or PET/CT and other multimodal imaging technologies, more comprehensive tumor information can be provided, improving diagnostic accuracy. Recently, trophoblast cell surface antigen 2 (Trop2)-based targeted molecular probes ^68^Ga-NOTA-RTD01 show promising diagnostic potential in preclinical pancreatic cancer models.^31^

### 2.3 Optical imaging

**Fig. 2:**
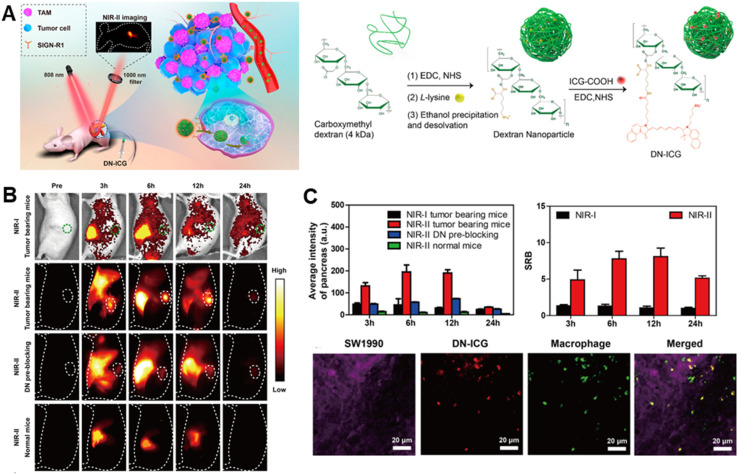
NIR II imaging based on diagnosis of PDAC. (A) Scheme of DN-ICG nanoprobes for NIR-II fluorescence imaging of TAMs in the pancreatic carcinoma microenvironment and synthetic scheme of DN-ICG: (B) *in vivo* NIR-I and NIR-II fluorescence imaging of DN-ICG in tumor-bearing and normal mice; (C) quantitative fluorescence signals, and SBR and immunofluorescent staining.

*In vivo* optical imaging exhibits non-radioactivity, non-invasiveness, high sensitivity and specificity, and real-time dynamic imaging advantages. Bioluminescence imaging (BLI) and fluorescence imaging (FI) are its two primary subtypes.^32^ FI uses a fluorescent reporter group for labeling, whereas BLI uses a luciferase gene to label cells or DNA. Although primarily used in preclinical studies, these techniques hold promise for intraoperative imaging, guiding surgeons during tumor resection Sensitive optical instruments are used to monitor living organisms' cellular activities and gene behaviors, to watch the development of diseases, tumor growth and metastasis, gene expression and response, and other biological processes in living animals. Optical imaging has limited tissue penetration, so research directions are geared towards combining different modalities. Recently, near-infrared (NIR) II fluorophores have deeper tissue penetration and can be coupled with ligands targeting PDAC-specific antigens (Fig. 2). Molecular imaging can significantly enhance surgical outcomes by providing real-time visualization of tumor margins and metastatic sites. Fluorescence-guided surgery (FGS) using tumor-specific probes can help achieve more precise resections, reducing the likelihood of residual tumor cells and improving survival rates.^33–39^

Molecular imaging also includes US, PAI and Raman optical imaging,^40^ which will not be covered in this paper as we focus on MRI as the core imaging modality.

## 3. Multimodality imaging

Tumor development is a dynamic, systemic, and complex process. This process involves the interaction of numerous molecular pathways and processes. A single imaging approach or scaling technique can't adequately resolve the intricacy of tumors. To gather complete and precise information on the lesion, a combination of imaging techniques with numerous modalities and characteristics is required. Multimodal molecular imaging is the use of two or more detection techniques to obtain additional information in diagnosis, therapy, and monitoring. Currently, multimodal molecular imaging is widely employed to improve medical research and practices.^41^

### 3.1 MRI/*in vivo* optical imaging

Optical Imaging (OI) offers excellent detection sensitivity but poor spatial resolution. MRI is noted for its superior tissue penetration and good anatomical resolution. However, it is significantly less sensitive. To compensate for each other's deficiencies, dual-modality fluorescence/magnetic resonance imaging (OI/MRI) technology combines OI's high sensitivity with MRI's high resolution to produce imaging reagents with optimal resolution, sensitivity, and depth penetration. This dual-modality imaging reagent is useful in clinical applications for detecting cancers and acquiring comprehensive information, offering a new approach for accurate tumor detection and treatment.

**Fig. 3:**
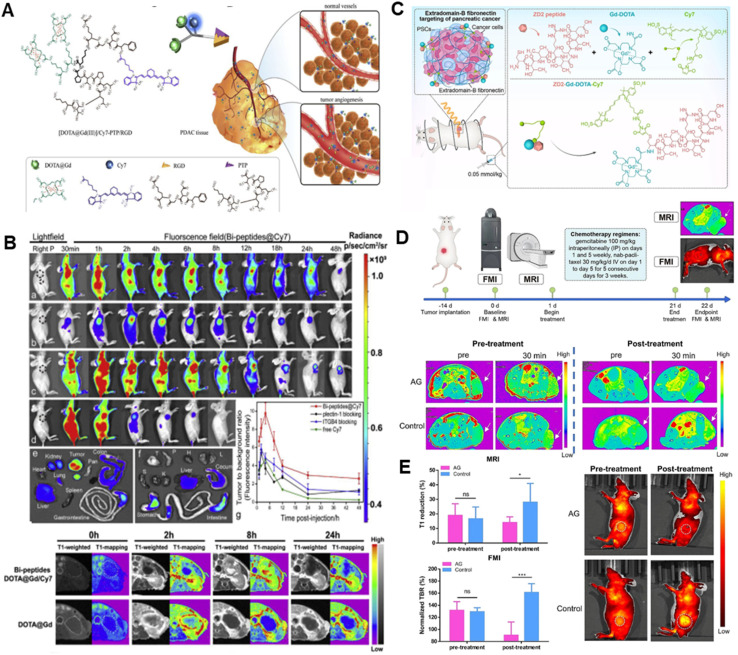
Magnetic resonance/near-infrared imaging of pancreatic cancer. (A) Schematic illustration of the bispecific probe targeting PDAC; (B) *in vivo* bio-distribution and dual-modality imaging of Gd-Cy7-PTP/RGD; (C) schematic structure of ZD2-Gd-DOTA-Cy7; (D) schematic illustration of the tumor model treatment strategy and therapeutic monitoring using a dual-modality imaging diagram and representative MRI images; (E) quantitative comparison and representative FMI images.

Glypican-1(GPC-1) is a cell surface glycan that is highly expressed in PDAC, based on which a bimodal imaging probe (Gd-Au-NC-GPC-1 (ref. 42)) was designed for the targeted detection of PDAC; the particles were able to efficiently target pancreatic cancer cells with a high expression of GPC-1, and an intense red fluorescence and a T_1_ image were detected 30 minutes after the injection into the mice. The UPAR-targeted MR/near-infrared fluorescent (NIRF) dual-modal probe (ATF-IO,^43^ DGL-U11 (ref. 44)) has good biocompatibility and imaging diagnostic capability. Researcher showed that 93% of pancreatic ductal adenocarcinoma cases are plectin-1 positive, and the specificity and sensitivity of plectin-1 in distinguishing malignant from benign lesions are 83% and 84%.A study reported a two-mode nanoparticle targeting plectin-1 (Plectin-SPION-Cy7).^45^ Both *in vitro* and *in vivo* data showed that nanoparticles targeting plectin-1 were highly accumulated in cancer cells/tissues but not in non-cancer cells/tissues. In a previous study, our team reported a plectin/integrin-targeted bispecific molecular probe (Gd-Cy7-PTP/RGD)^46^ (Fig. 3A and B) applied to e PDAC diagnosis, which guided surgical resection by MRI/NIRF dual-modality imaging. These results suggest that targeted plectin-1 fluorescence and magnetic resonance bifunctional nanoparticles can be used for pancreatic cancer diagnosis.In addition, photomagnetic probes targeting Survivin^47^ have also shown enhanced targeting. They provide a promising strategy for the diagnosis of pancreatic precancerous lesions. Upconversion nanoparticles (UCNPs) are a type of nanomaterial capable of converting low-energy light into high-energy light. Researchers^48^ created upconversion nanoparticle micelles (UPGs) targeting CD326 and imaged them in a human pancreatic cancer xenograft mouse model, demonstrating CD326's excellent active targeting ability.

The advancement of molecular and functional imaging technologies enhances the identification of cancer lesions. It offers a potent instrument for real-time monitoring of treatment efficacy and adjustment of personalized therapeutic regimens. Bimodal contrast agents targeting MUC4 demonstrated promising imaging results.^49^ To monitor the chemotherapy response, Wang created a bimodal molecular imaging probe (MN-EPPT^50^) that targets the underglycosylated mucin 1 (uMUC1). The group receiving gemcitabine treatment had less MN-EPPT accumulation in their tumors, which suggests a significant decrease in the expression of the uMUC1 antigen. This finding supported the hypothesis that the uMUC1 antigen was down-regulated during chemotherapy and suggested that this imaging technique might help predict how well PDAC treatments would work. Zhang *et al.*^51^ also developed a bimodal imaging platform using an imaging probe (ZD2-Gd-DOTA-Cy7) to non-invasively, dynamically and quantitatively assess PDAC chemotherapy-induced fibrosis by NIR fluorescence molecular imaging and MRI (Fig. 3C–E).

### 3.2 MRI/MPI/*in vivo* optical imaging

Magnetic Particle Imaging (MPI) is a new *in vivo* bioimaging technology that has emerged in recent years. Unlike standard anatomical imaging, it offers ultra-high sensitivity, no background interference, and is independent of scanned tissue depth. It is also devoid of ionizing radiation, allowing for high-definition thermographic imaging.^52^ However, its spatial resolution is limited, and it lacks anatomical information. As a result, in clinical practice, we have to keep trying to integrate the advantages of different imaging techniques to collect more exact anatomical features and provide more comprehensive and in-depth information for PDAC diagnosis and therapy.

Zhang *et al.*^53^ combined a targeted plectin-1 peptide and a near-infrared dye IRDye800CW, with superparamagnetic iron oxide nanoparticles (PTP-Fe_3_O_4_-IRDye800CW) for tri-modal imaging of PDAC targeting. Subcutaneous and *in situ* PDAC mice models were created to allow for dynamic and quantitative surveillance of PDAC tumors using FI, MPI, and MRI. The findings showed that the particles performed admirably in terms of trimodal imaging and targeted targeting. The particles showed higher specificity, homogeneous distribution, and retention in tumors for up to 7 days compared to controls. Another study encapsulated iron nanoparticles and the optical dye indocyanine green (ICG) into liposomes made of cationic sphingomyelin.^54^ The particles were also assessed by MRI, optical imaging, magnetic particle spectroscopy (MPS), and photoacoustic imaging (PAI).

### 3.3 Others

Emphasis is being placed on different combinations of different forms of multimodal imaging techniques. For instance, a multimodal imaging agent (AuNR-SiO_2_-Gd NPs^55^) combining MRI, CT, and PAI has demonstrated good imaging results, combining the advantages of high soft tissue contrast of MRI, the spatial resolution of CT, and the high sensitivity and molecular specificity of PAI. Peptide-22-Cy7,^56^ a probe targeting the LDLR receptor, uses the high spatial resolution and contrast of photoacoustic imaging to detect tumor lesions of approximately 4 mm pre-operation. Experiments^57,58^ showed that PET/NIRF/CLI was also useful for identifying PDAC lesions and guiding surgical procedures. Additionally, SPECT-CT, a handheld gamma camera, and MRI were combined to provide combined imaging modalities.^59^

To summarize, multimodal molecular medical imaging technology allows for precise tumor imaging by combining molecular probes with various signal emissions with tumor-specific ligands. Multiple sophisticated probes catch and integrate the released signals, allowing for high-resolution tumor imaging. This multimodal imaging strategy not only provides information on the tumor's anatomical structure, but also on its functional and metabolic state, providing an important foundation for early diagnosis, grading and staging, efficacy assessment, and prognosis determination. It allows us to comprehend the biological properties of tumors better, offering accurate advice for tailored diagnosis and treatment, which is the future of medical imaging advancement.

## 4. Advances in nanomedicine for pancreatic cancer theranostics

The application of nanotechnology in molecular imaging agents is quickly evolving, resulting in significant achievements in preclinical research and clinical translation. The utilization of nanoparticles as carriers, as well as the precise alteration of small molecules, peptides, antibodies, and other biomolecules, results in excellent targeting and biocompatibility, significantly increasing the sensitivity and specificity of molecular imaging technology. With the advancement of molecular imaging technologies, several significant study findings have been obtained. These accomplishments include not only the creation of new imaging agents, but also the optimization of imaging procedures, the investigation of clinical applications, and a thorough understanding of disease causes.

### 4.1 Therapeutic nanoparticles

Nanomedicines, as sub-micron scale carrier materials, have become an essential component of drug delivery systems.^64^ The continual advancement of nanotechnology has transformed the diagnosis and treatment of PDAC. New nanoformulations have been shown to improve the activity of conventional chemotherapeutic agents, such as gemcitabine, and new antitumor drugs, protecting them from degradation, improving their selectivity, solubility and bioavailability, and reducing their side effects. Liposomes, polymers, micelles, solid (lipid) nanoparticles, and antibodies are some of the most frequent nanomaterials employed in medicine.^65^ Several important issues must be considered while designing nanoscale drug delivery systems to ensure their safety, efficacy, and practicality.^66,67^ Nanoscale drug delivery systems can be designed carefully to be safe and efficient, providing new tactics and instruments for disease treatment.^68^

Because undifferentiated tumor tissue frequently features malfunctioning lymphatic arteries and a “leaky” vascular system, nanoparticles can “passively” collect within the tumor tissue, allowing the active ingredient to be preferentially deposited at the target site. The mechanism referred to as the “enhanced permeability and retention effect (EPR)^69^” enables therapeutic drugs to be delivered to tumor cells *via* nanoparticles with minimal negative effects on normal tissue. However, the highly heterogeneous and complicated biology of the tumor microenvironment in PDAC decreases drug delivery efficiency, requiring the development of more effective active targeting techniques to identify and accumulate nanocarriers in tumor tissues specifically. In recent years, with a better understanding of PDAC's pathophysiology and the progressive reveal of its microenvironment, researchers have conceived and produced many “active targeting” drug delivery systems. These include targeting the transferrin receptor,^70^ EGFR,^71–73^ integrin receptor,^74^ and uPAR^75,76^ on tumor cell surfaces. There are also ways to target pancreatic stellate cells,^77^ tumor stem cells,^78,79^ and other tumor-related cell types. These strategies aim to enhance medication concentrations in tumor tissues and overcome biological barriers in the tumor microenvironment, improving the efficacy of treatment. These unique targeted delivery systems are intended to provide patients with more effective and tailored therapy alternatives.

**Fig. 4:**
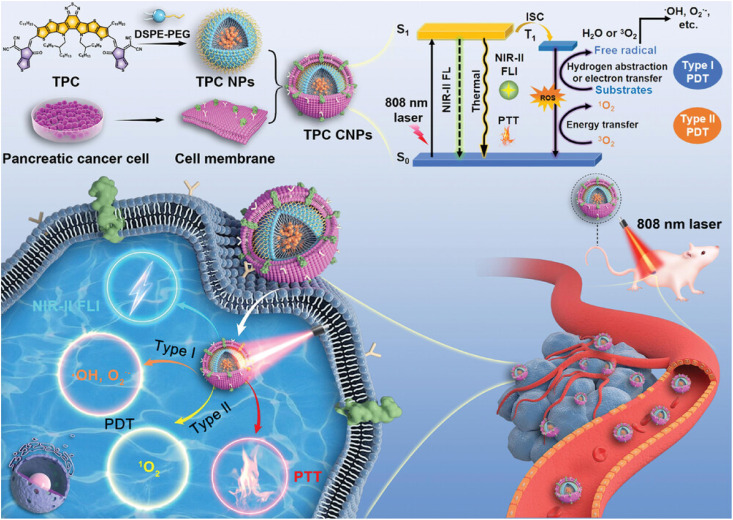
NIR-II-emissive organic nanomedicine with biomimetic engineering for bioimaging targeting and multiple phototherapies of pancreatic tumors.

The exact release of nanomedicines in the tumor microenvironment is difficult. Acidic pH, higher glutathione (GSH), and hypoxia are PDAC microenvironmental factors that contribute to therapeutic resistance by influencing cancer metabolic processes and generating an environment that promotes tumor cell survival.^80^ Researchers have used physiological and physical properties (*e.g.* pH,^81^ GHS,^82^ ROS,^83^ and hypoxia^84^) to optimize drug carrier design, regulate drug release mechanisms, and create a variety of smart-responsive nanomedicines. It is possible to attack cancer cells precisely while avoiding damage to normal tissues. The distribution, metabolism, and clearance pathways for nanocarriers can have an impact on their efficacy and safety. To reduce potential toxicity, nanocarriers must remain at the tumor site for an extended period of time and be successfully eliminated once the therapeutic impact has been achieved. To assure the efficiency and durability of nanoparticles, researchers used several targeting strategies,^85^ such as local injectable therapy,^86,87^ magneto-thermal/photo-guided therapy,^88–90^ and alteration of the “don't eat me” signal.^91^ Wang *et al.* introduced a groundbreaking NIR-II-emissive organic nanomedicine TPC, enhanced through biomimetic engineering, designed for high-contrast targeted bioimaging and multifaceted phototherapies of pancreatic tumors (Fig. 4). This innovative nanomedicine showcased remarkable tumor-targeting abilities, facilitating high-resolution NIR-II bioimaging and exceptional phototheranostic performance. Their work offers a promising pathway for the advancement of targeted and high-efficiency theranostic strategies.^92^ Furthermore, biocompatible materials and designs are required for nanocarriers in order to prevent undesired immunological reactions or inflammation. Additional significant factors that require careful consideration are the possible toxicity and long-term safety of nanocarriers. Researchers have successfully addressed the technological benefits of endogenous membranous carriers, such as longer circulation time, immunological evasion, adhesion, and homologous targeting, by employing bionic carriers such as cell membranes and homologous exosomes from tumor cells.^93,94^

### 4.2 Multimodal theranostic nanoparticles

Surgery, chemotherapy, radiotherapy, immunotherapy, anti-stromal therapy, photo/acoustic-dynamic therapy, gene therapy and multimodal combination therapy for PDAC have all seen significant advancements thanks to nanomedicine. Previously, much research has focused on using conventional molecular biology testing techniques to measure variations in the levels of protein expression as well as the transcriptional and translational activities of oncogenes both before and after medication therapy. However, due to the frequent fragmentation, these data are unable to accurately depict the “dynamic” features of the drug delivery system, including its temporal and spatial dispersion during *in vivo* metabolism. In addition, the crucial timing of drug delivery systems' metabolism, therapeutic effects, and related cellular and molecular processes throughout treatment cannot be revealed by these methods. Thus, with the use of cutting-edge visual imaging tools, it is imperative to carry out thorough investigations on the mechanisms of drug delivery system enrichment, drug release, distribution, and efficacy assessment in tumors to provide more thorough information to optimize therapeutic options. Targeted imaging techniques like two-photon imaging, FI, MRI, SPECT, PET, CT, PAI, and US have been made possible by the present use of multifunctional nanoparticles.^95^ According to nanotechnology, many therapeutic particles can be fused and delivered precisely where they should be used in nanotargeted therapies. In similar terms, we may consider packing therapeutic and diagnostic particle packages into a single carrier to fulfill the complementary objectives of monitoring and treating.^96^

Qiu *et al.*^62^ designed a GPC1-targeted, multifunctional gold nanocarrier loaded with gemcitabine for NIRF/MRI imaging and treatment against PDAC. Experiments conducted *in vivo* and *in vitro* displayed that the nanocarrier's ability to produce significant amounts of reactive oxygen species in response to light might inhibit the growth of tumors. A fluorescent iron oxide and gemcitabine-encapsulated nanosphere targeting human epidermal growth factor receptor 2 (HER-PGFIO),^63^ combining chemotherapy, thermotherapy, and MRI imaging for PDAC treatment and imaging, dynamically reflected its accumulation at the tumor site and therapeutic changes. Qu *et al.*^97^ developed a nanomaterial that enhances the effectiveness of photodynamic therapy and immunotherapy. It not only makes multimodal imaging of PDAC possible, but enhances immunotherapy's effectiveness by remodeling the TME and immunogenic cell death(ICD); Moreover, pancreatic cancer cells' antigenic appearance, structure, and associated functions are inherited by CNP@folfirinox,^93^ a unique nanoparticle drug delivery platform that displays exceptional homologous homing to tumor tissues and deep penetration capacity as well as fewer negative consequences. Using BLI and MRI, the significant therapeutic efficacy and biodistribution of the nanoparticles were evaluated in mouse models.

Multimodal imaging plays a crucial role in the development of nanotherapeutic drugs. This advanced imaging technology allows researchers to gain an in-depth comprehension of the dynamic distribution, mechanism of action, and therapeutic effects of nanomedicines in tumor tissues from a variety of angles. This strong support for precision medicine and tailored treatment plans is anticipated to greatly enhance both the quality of life for patients and the efficacy of tumor treatment. The role of multimodal imaging technology in tumor therapy will grow as it continues to be developed and optimized.

### 4.3 Nanoparticle-based immunotherapy of pancreatic cancer

Nanoparticle-based immunotherapy such as cancer vaccines, chimeric antigen receptor T cell therapy and immune checkpoint inhibitors hold significant promise for the treatment of pancreatic cancer by enabling precise delivery and enhanced efficacy of immunotherapeutic agents. By addressing the unique challenges posed by the tumor microenvironment and advancing combination therapies, this approach has the potential to improve outcomes for patients with pancreatic cancer.

Chimeric antigen receptor T (CAR-T) cell therapy is regarded as a highly effective treatment for relapsed or resistant tumors, particularly hematological malignancies.^98^ The severe cytokine release syndrome, as well as other toxicities, limits clinical use and therapeutic efficacy.^99^ There is a need for tools that can dynamically and quantitatively evaluate the effective timing, therapeutic efficacy and safety of their treatments, and track key processes such as the characteristics of *in vivo* distribution and the timing of onset of action after the transfusion of the overall T cells in the body. The researchers^100^ used iron oxide nanolabelling to label CAR-T cells, and they achieved multimodal *in vivo* monitoring of T cells using MRI, (photoacoustic tomography) PAT, and MPI at the same time. Another researcher^101^ labelled T cells with NIRF silica nanoparticles and ^89^Zr nuclides, leading in a bimodal PET/NIRF nanolabelling that was subsequently employed in a rat ovarian peritoneal carcinomatosis model to track the *in vivo* distribution of CAR-T cells. The scientists point out that the imaging technique can also be used on different cancer models. Due to limited therapeutic efficacy in solid tumors, CAR-T multimodal molecular tracer technology has not been successfully applied to PDAC. Efficient imaging and adaptation of therapeutic strategies will expand the use of CAR-T cell therapies in solid tumors, and the development of dynamic imaging platforms is critical for monitoring the efficacy and toxicity of novel CAR-T cell therapies. Moreover, a MR-CA nanoprobe-based MRI could monitor hypoxia reduction by tumor normalization treatments, which permits visualizing pancreatic tumors that will respond to immune checkpoint blockade therapy, enhancing the response rate.^102^

### 4.4 Potential applications of nanoparticles

The development of appropriate *in vivo* stem cell tracers to track the survival, migration, proliferation, and differentiation of stem cells after transplantation *in vivo* is critical for the clinical translation of stem cell therapy.^103^ Huang *et al.*^104^ designed a nanoparticle for dual-modal tracing of stem cells by CT/NIRF (AA@ICG@PLL), which achieved 21-day tracing of stem cells *in vivo*; Lim *et al.*^105^ synthesized nanoparticles encapsulating Fe_3_O_4_/Cy5.5, which were used to label stem cells using the click reaction of azide groups with alkyne groups, and were used for tracing stem cells in MRI/NIRF bimodal imaging of stroke in rats; likewise, MPI's high sensitivity, minimal signal attenuation, and capacity to measure the cell count are important benefits for stem cell tracers;^106–108^ Gene therapy has progressed from the laboratory to a range of clinical applications, and it is essential to non-invasively monitor endogenous gene expression and efficacy in real-time *in vivo*, which is one of the current hotspots of molecular imaging research. Currently, the most common imaging techniques are FI and nuclear reporter gene imaging.^109,110^ Moreover, multimodal imaging is an effective method for tracing tumor exosomes, improving understanding and monitoring of exosome biological behavior by combining the benefits of different imaging modalities.^111–114^

In biomedical applications of nanomaterials, biocompatibility remains a central concern, particularly for heavy metal-containing nanomaterials. Enhancing their biosafety requires systematic optimization across the following key aspects: (1) selection of inherently biocompatible base materials to minimize intrinsic toxicity.^115,116^ (2) Precise surface chemistry modulation, such as biomolecule functionalization (*e.g.*, proteins, polysaccharides, or nucleic acids), to improve stealth and targeting.^117^ (3) Optimization of size and morphology to reduce cellular uptake barriers and enhance biodistribution. (4) Controlled degradation properties to prevent long-term accumulation and associated toxicity. (5) Development of multidimensional physiochemical-biological characterization systems for comprehensive safety evaluation. Notably, nanomaterials may still pose risks such as oxidative stress, mitochondrial dysfunction, immunogenicity, organ-specific accumulation, and genotoxicity.^118^ To address these challenges, researchers have engineered environmentally responsive “smart” carriers (*e.g.*, tumor microenvironment-triggered drug release systems), which significantly improve targeting efficiency while reducing systemic toxicity.^119^

Nanoprobe technology is rapidly transitioning from a single functional modality into a multifunctional, multimodal, and intelligent imaging multiplex. Despite considerable advances in this field, concerns such as biocompatibility, target selectivity, and therapeutic coordination remain to be addressed. Overall, the use of nanotechnology in the field of molecular imaging agents is propelling the advancement of molecular imaging technology to a new level. Some of the technologies are still in their early stages of use in PDAC, and there are ongoing research challenges as well as potential research hotspots. In particular, multifunctional nanomaterials are gaining importance in precision medicine.

## 5. Challenges and prospects for clinical translation

In summary, nanomedicine offers a versatile and powerful toolkit for the theranostics of pancreatic cancer, with the potential to significantly improve diagnosis, treatment, and patient outcomes. Clinical translational research on molecular imaging probes and targeted tracers for PDAC remains in its early stages, with limited published clinical trial data and most candidate molecules still in preclinical development. Notably, significant progress has been made in nuclear medicine, where radionuclide probes targeting fibroblast activation protein (FAP) and prostate-specific membrane antigen (PSMA) have shown promising clinical potential. Clinical trial data demonstrate that diagnostic and therapeutic agents such as ^18^F-PSMA PET/CT,^120^^68^Ga-FAPI-46,^121^ and ^90^Y-FAPI-46 (ref. 122) exhibit excellent targeting specificity and clinical efficacy in PDAC management. Building on these encouraging results, future efforts should focus on accelerating the translation of additional candidate molecules from bench to bedside and conducting larger-scale clinical trials to validate their safety and therapeutic value.

However, translating these advances from the laboratory to the clinic requires addressing several biological and regulatory challenges. Dense stromal tissue and poor vascularization in pancreatic tumors pose significant challenges for nanoparticle delivery. This stroma, composed of fibroblasts, an extracellular matrix, and immune cells, creates a physical barrier that impedes the penetration and distribution of nanoprobes within the tumor. Consequently, achieving effective delivery of therapeutic and diagnostic agents to the tumor cells becomes difficult. One approach involves using stromal-depleting agents, such as hyaluronidase, which can degrade components of the extracellular matrix and reduce stromal density, thereby improving nanoprobe access to tumor cells. Additionally, nanoparticles can be engineered with surface modifications, such as polyethylene glycol (PEG) coatings, to improve their circulation time and enhance their ability to penetrate the tumor stroma. Targeting specific receptors overexpressed in the stroma, such as integrins, can also facilitate more effective delivery of nanoprobes. Preclinical research on nanoscale therapeutic diagnostic particles is currently underway, focusing on evaluating the efficacy of novel particles through various animal models such as subcutaneous and *in situ* pancreatic cancer models which use pancreatic cancer cell lines, patient-derived xenografts (PDX), and genetically-engineered mice. Numerous studies have been conducted using different modelling methods; however, a standardized criterion for validating the efficacy of these models has not yet been established. It is evident that existing animal models do not fully replicate the *in vivo* state of PDAC. Therefore, the development of animal models that better reflect the heterogeneity and histological anatomy of PDAC is imperative for successful translation of these nanoscale particles to clinical settings.

The time point at which a meaningful signal difference or a therapeutic effect occurs in MRI images after particle injection in an animal model ranges from within an hour to a few days, as indicated by the studies discussed previously. Given the importance of these findings for future clinical applications, it is imperative to establish a more standardized approach to the timing of imaging. Additionally, ensuring a uniform standard in imaging practices is essential for controlling variability across studies. Research progress can be significantly impeded by low spatial resolution and sensitivity. Molecular imaging equipment is expensive, and the disparity in equipment levels across research institutions, such as MRI ranging from 1.5T to 11.7T or higher, can lead to inconsistent study outcomes. Therefore, fostering collaboration between the medical and industrial sectors is critical for promoting standardized imaging practices in the future.

MRI provides detailed anatomical and functional information, but its application in molecular imaging remains limited due to inherently low sensitivity, significantly inferior to that of PET (picomolar level).^123^ To overcome this “low sensitivity *vs.* high resolution” trade-off, molecular MRI should advance in the following key areas: the development of nanoprobes (*e.g.*, ultra-small iron oxide particles^124^) with high relaxation rates, low toxicity, and long cycle times, combining physical means such as hyperpolarization and quantum sensing^125,126^ to increase the detection limit, and achieving responsive imaging of the tumor microenvironment (*e.g.*, pH and hypoxia) to guide targeted therapies.^127^

The integration of artificial intelligence (AI) in the diagnosis of pancreatic cancer represents a significant leap forward in medical imaging and diagnostic accuracy.^128^ AI algorithms, particularly those employing machine learning and deep learning techniques, have shown promise in enhancing the early detection and precise characterization of pancreatic tumors.^129^ By analyzing vast amounts of imaging data from modalities such as CT, MRI, and PET scans, AI systems can identify subtle patterns and anomalies that may be indicative of early-stage pancreatic cancer, often overlooked by human radiologists.^130^ Preoperative deep learning models can also analyze lymph node metastasis^131^ and predict postoperative survival based on clinical and imaging data.^132^ Integrating AI with traditional diagnostic tools not only enhances the precision of pancreatic cancer diagnosis but also opens up new possibilities for personalized treatment planning, ultimately improving patient outcomes. In addition, AI technology has demonstrated significant advantages in the field of medical image analysis, which not only enables automated and accurate segmentation of lesions, but also effectively improves the image signal-to-noise ratio and resolution through advanced deep learning algorithms, thus significantly improving image quality.^133–135^

## 6. Conclusion and prospects

As a malignant tumor with high therapeutic difficulty and a poor prognosis, the medical field has always faced significant challenges in detecting and treating PDAC. Despite remarkable advances in medical research in recent years, there has been no significant improvement in patient survival and prognosis due to the complexity of the PDAC microenvironment, congenital and acquired drug resistance, and the desert-type immune microenvironment. Currently, alternatives to standard therapy are limited and confront numerous clinical hurdles. In this paper, we summarized the developments in PDAC molecular imaging research and highlighted the potential for improving PDAC diagnostic and treatment outcomes through the use of multimodal and multimodal therapeutic combinations. It is also significant to point out how nanotechnology is helping to integrate multimodal molecular imaging with diagnosis and treatment. We have good reason to anticipate that multimodal molecular imaging technology, with its ongoing development and optimization, will become even more important in clinical diagnosis and treatment in the future. It will offer novel approaches for early diagnosis, targeted therapy, and efficacy evaluation of PDAC.

**Fig. 5:**
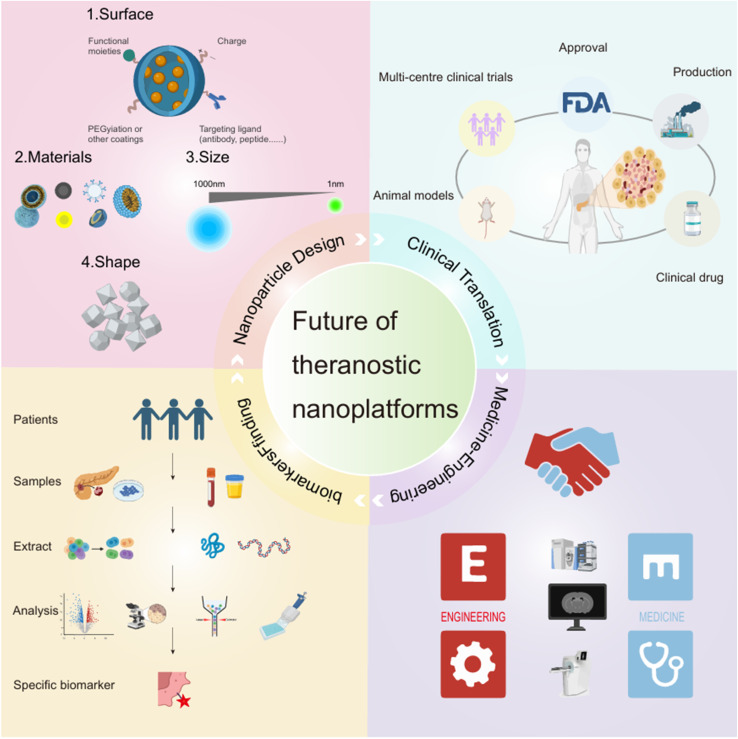
Future directions for theranostic nanoparticles.

However, both particular molecular imaging probes and multifunctional nanoparticle medications confront enormous challenges when it comes to industrialization, standardization, and clinical translational approval. Future studies should focus on specific clinical difficulties, validate the safety and usefulness of these probes in clinical applications, and investigate their creative usage in the treatment of PDAC. It is also critical to create novel preclinical models that better reflect medication distribution, PDAC heterogeneity, and therapy response. In the future, advancements in basic research, biomarker discovery, design modification of NPs, upgrading of imaging equipment and AI technology, and enhancement of clinical translation efforts are expected to bring more effective and precise treatment options to patients with PDAC (Fig. 5). Research on personalized treatment for PDAC is ongoing, and the incorporation of advances in nanomedicine and nanotechnology into PDAC diagnosis through a combination of novel therapies is anticipated to significantly enhance the survival rate and quality of life of PDAC patients.

## Data availability

No primary research results, software or code have been included and no new data were generated or analysed as part of this review.

## Author contributions

Xun Hu, Zihua Wang and Yuting Zhu wrote the manuscript. Hao Yan, Xinming Zhao and Qian Wang conceived the study and also revised the paper. All authors approved the final version.

## Conflicts of interest

The authors declare no conflict of interest.
